# Preoperative prediction of lymph node metastasis using deep learning-based features

**DOI:** 10.1186/s42492-022-00104-5

**Published:** 2022-03-07

**Authors:** Renee Cattell, Jia Ying, Lan Lei, Jie Ding, Shenglan Chen, Mario Serrano Sosa, Chuan Huang

**Affiliations:** 1 Department of Biomedical Engineering, Stony Brook University, NY 11794 Stony Brook, USA; 2 Department of Radiation Oncology, Renaissance School of Medicine, Stony Brook University, Stony Brook, NY 11794 USA; 3 Program in Public Health, Stony Brook Medicine, Stony Brook, NY 11794 USA; 4 Department of Medicine, Northside Hospital Gwinnett, GA 30046 Lawrenceville, USA; 5 Department of Radiation Oncology, Medical College of Wisconsin, Milwaukee, WI 53226 USA; 6 Institute of High Energy Physics, Chinese Academy of Sciences, Beijing, 100049 China; 7 Department of Radiology, Renaissance School of Medicine, Stony Brook University, Stony Brook, NY 11794 USA

**Keywords:** Deep learning, Radiomics, Prediction model, Lymph node metastasis, Breast cancer

## Abstract

Lymph node involvement increases the risk of breast cancer recurrence. An accurate non-invasive assessment of nodal involvement is valuable in cancer staging, surgical risk, and cost savings. Radiomics has been proposed to pre-operatively predict sentinel lymph node (SLN) status; however, radiomic models are known to be sensitive to acquisition parameters. The purpose of this study was to develop a prediction model for preoperative prediction of SLN metastasis using deep learning-based (DLB) features and compare its predictive performance to state-of-the-art radiomics. Specifically, this study aimed to compare the generalizability of radiomics vs DLB features in an independent test set with dissimilar resolution. Dynamic contrast-enhancement images from 198 patients (67 positive SLNs) were used in this study. Of these subjects, 163 had an in-plane resolution of 0.7 × 0.7 mm^2^, which were randomly divided into a training set (approximately 67%) and a validation set (approximately 33%). The remaining 35 subjects with a different in-plane resolution (0.78 × 0.78 mm^2^) were treated as independent testing set for generalizability. Two methods were employed: (1) conventional radiomics (CR), and (2) DLB features which replaced hand-curated features with pre-trained VGG-16 features. The threshold determined using the training set was applied to the independent validation and testing dataset. Same feature reduction, feature selection, model creation procedures were used for both approaches. In the validation set (same resolution as training), the DLB model outperformed the CR model (accuracy 83% vs 80%). Furthermore, in the independent testing set of the dissimilar resolution, the DLB model performed markedly better than the CR model (accuracy 77% vs 71%). The predictive performance of the DLB model outperformed the CR model for this task. More interestingly, these improvements were seen particularly in the independent testing set of dissimilar resolution. This could indicate that DLB features can ultimately result in a more generalizable model.

**Supplementary Information:**

The online version contains supplementary material available at 10.1186/s42492-022-00104-5.

## Introduction

Breast cancer increases in stage and severity as it metastasizes to axillary lymph nodes [1]. Lymph node involvement increases the risk of recurrence and acts as a prognostic indicator, with the survival rate of node-positive patients being up to 40% lower than node-negative patients [2–6]. As a result, lymph node status is critical for diagnosis, prognosis, and monitoring of treatments [7].

Although lymph node management has become less invasive with the use of sentinel lymph node (SLN) biopsy as opposed to full axillary lymph node dissection, significant side effects including shoulder dysfunction, lymphedema, and nerve damage were still observed in as much as one-fourth of patients [8, 9]. Moreover, studies have reported > 70% of biopsied SLNs are negative [8], indicating that such procedure is unbeneficial and potentially harmful to a significant amount of breast cancer patients. Accurate non-invasive assessment of nodal involvement therefore is valuable in cancer staging, surgical risk, and financial cost reduction.

Breast cancer is an area of peaked interest for the combination of radiomics and artificial intelligence, with clinical impact possible as both a diagnostic and prognostic tool [10]. One such task is the development of a predictive model for non-invasive staging of the axillary lymph nodes as an alternative to SLN biopsy. Nomograms and radiomic pipelines have been used to predict SLN status with promising results [9, 11–19]. However, conventional radiomics (CR) has several disadvantages. For instance, the robustness of the conventional hand-crafted radiomic features is variable based on changing parameters, including pixel size, region-of-interest (ROI) delineation, and signal-to-noise ratio [20]. Deep learning has the potential to serve as a more powerful tool to overcome these issues as shown in several studies [21–26]. Moreover, deep learning is capable of learning high-level and task-adaptive image features [27]. It enables direct feature extraction from multiple levels without explicit definition and can provide a higher level of feature abstraction [28]. However, deep learning requires a large training data size to obtain a generalizable and functional classification model. Fortunately, studies have demonstrated that initial features extracted by deep learning network are largely similar to CR, since they both detect edges, ripples, and various other textures prior to observing more complex features [29–31]. Thus, it is possible to use features identified by a pre-trained deep learning network as an alternative to hand-crafted features used in CR.

The purpose of this study was to develop a DLB feature prediction model for preoperative prediction of SLN metastasis and compare its predictive performance to state-of-the-art CR. Specifically, this study aimed to compare the generalizability of CR vs DLB features in an independent testing set of dissimilar resolution.

## Methods

**Fig. 1:**
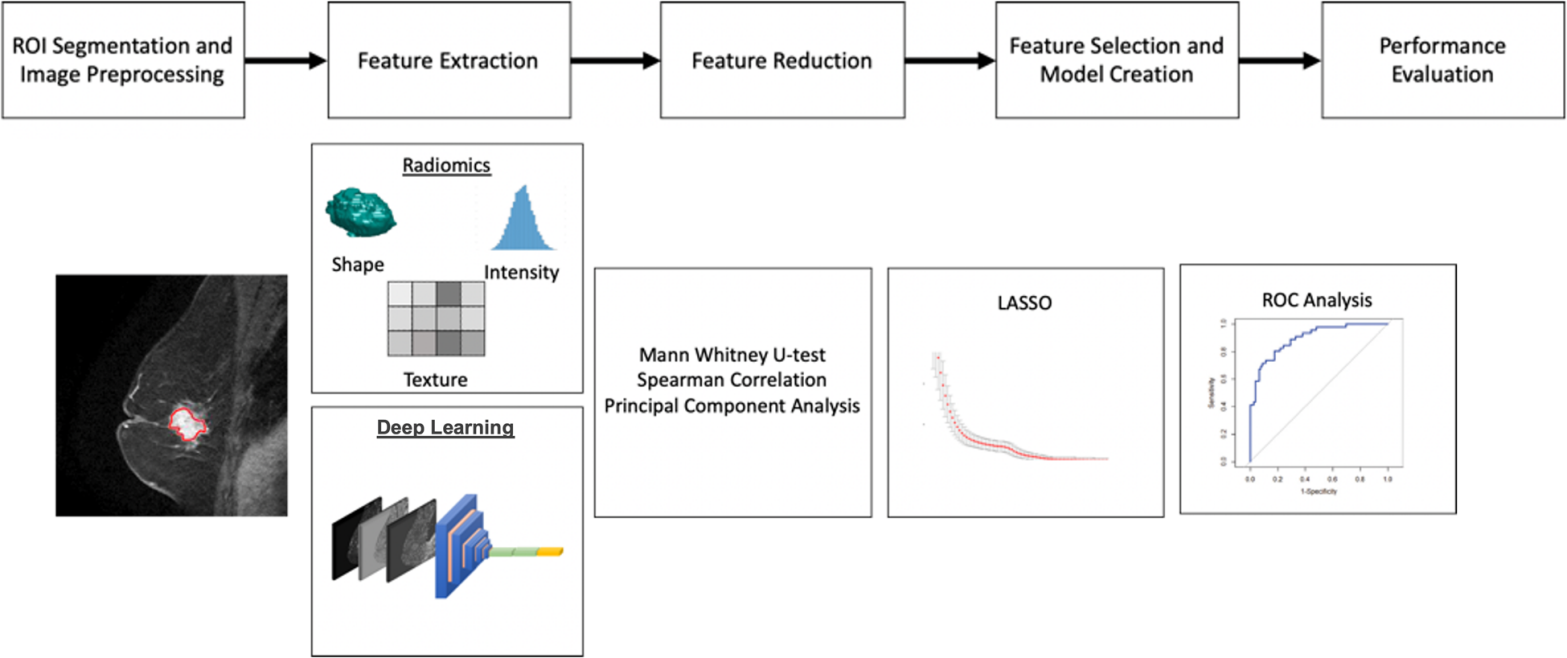
Schematic representation of pipeline for feature extraction, reduction, and model creation. The CR pipeline and the pipeline using deep learning-based (DLB) features are only different in their feature extraction step. All other steps remain identical. LASSO: Least absolute shrinkage selection operator; ROC: Receiver operating characteristic

Figure 1 shows the general pipeline used in this work.

### Study population

The dataset used in this study is an expansion of that described in previous publication [13]. Briefly, data for this institutional review board-approved retrospective study collected images from June 2013 to June 2017. Inclusion criteria were patients that had (1) preoperative dynamic contrast enhanced (DCE)-magnetic resonance imaging (MRI), (2) diagnosis of invasive breast cancer by histopathology, (3) SLN biopsy result, and (4) no neoadjuvant chemotherapy. Exclusion criteria were patients that had (1) no SLN biopsy result, (2) very small tumor ROI (less than 64 voxels), or (3) MRI after neoadjuvant chemotherapy. After inclusion/exclusion criteria, a sample of 198 patients (67 positive SLNs and 131 negative SLNs) was used in this study. Of those 198 subjects, 163 had an in-plane resolution of 0.7 × 0.7 mm^2^; that 163 subject cohort was randomly divided into two independent subsets: a training set (approximately 67%, 109 patients with 37 positive SLNs) and a validation set (approximately 33%, 54 patients with 18 positive SLNs).

The remaining 35 subjects (35 patients with 12 positive SLNs) with a different in-plane resolution (0.78 × 0.78 mm^2^) were treated as an independent testing set with dissimilar resolution to test the generalizability of the predictive models for imaging data acquired with slightly different resolution. Given that radiomics has been shown to have limited generalizability, an independent testing set of dissimilar resolution will more rigorously assess this potential of the predictive model.

Clinical data collected for this study included whether the tumor was confined to the upper inner quadrant, multifocality, age, pathological type, tumor grade, molecular subtype, and lymphovascular invasion.

### MRI examination

The MRI examinations were all performed using a dedicated 8-channel breast coil on 1.5 T GE Signa (GE Healthcare, Wauwatosa). The sequence of interest in this study was the DCE series; sagittal VIBRANT multiphase sequence was acquired with the following parameters: repetition time (TR) = 4.46–7.80 ms; echo time (TE) = 1.54–4.20 ms; flip angle = 10°; matrix = 256 × 256; slice thickness = 2 mm. I.V. contrast agent was Magnevist (Schering, Berlin), injected at a dose of 0.2 mL/kg at a rate of 2 mL/s, followed by 20 mL saline flush. Five phases were acquired: one pre-contrast and four post-contrast images. Patients with pixel sizes of 0.7 × 0.7 mm^2^ were split into training and validation cohorts. Patients with pixel sizes of 0.78 × 0.78 mm^2^ were separately analyzed in an independent testing set. This analysis allows for the clinically practical reality that it is knowingly difficult to standardize pixel size, which may need to be adjusted based patient specific variable (e.g., size of the patient).

### Map calculation

**Fig. 2:**
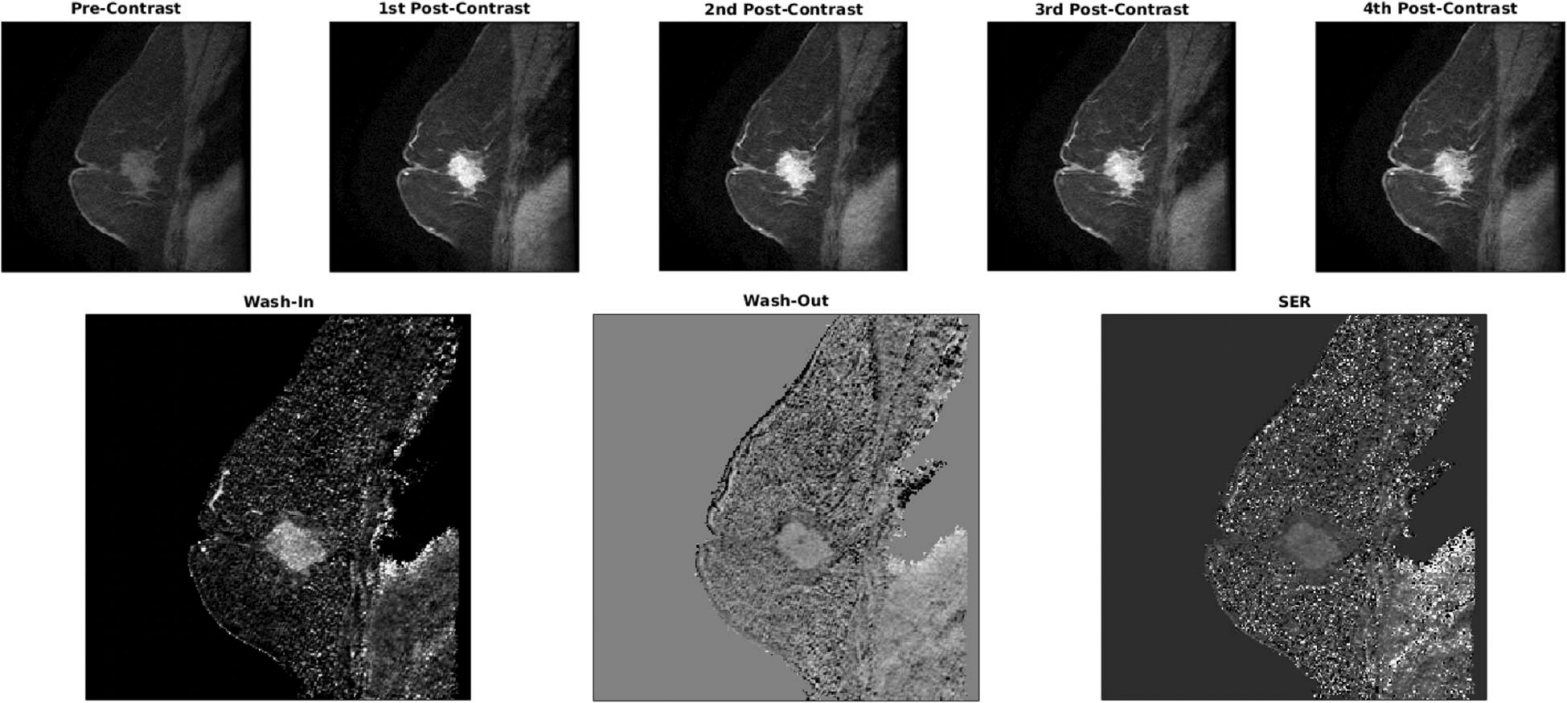
Representative image of pre- and post-contrast images of an in-breast tumor and the calculated Wash-In, Wash-Out, and SER maps. Equations for map calculation: Wash-in map ((S_1_-S_0_)/S_0_) × 100%, Wash-out map ((S_1_-S_4_)/S_1_)) × 100%, SER map ((S_1_-S_0_)/(S_4_-S_0_)) × 100%, where S_0_, S_1_, and S_4_ are the pre-contrast, first postcontrast, and fourth (the last) post-contrast images, respectively

Reducing the effect of varying TR and TE, three ratio maps were used: wash-in maps ((S_1_-S_0_)/S_0_) × 100%, wash-out maps ((S_1_-S_4_)/S_1_)) × 100%, and signal enhancement ratio (SER) maps ((S_1_-S_0_)/(S_4_-S_0_)) × 100%, where S_0_, S_1_, and S_4_ are the pre-contrast, first post-contrast, and fourth (the last) post-contrast images, respectively. These maps are independent of the original MR signal intensity and capture the behavior of contrast enhancement in the tissue. Representative image and calculated kinetic maps are shown in Fig. 2.

### Segmentation

**Fig. 3:**
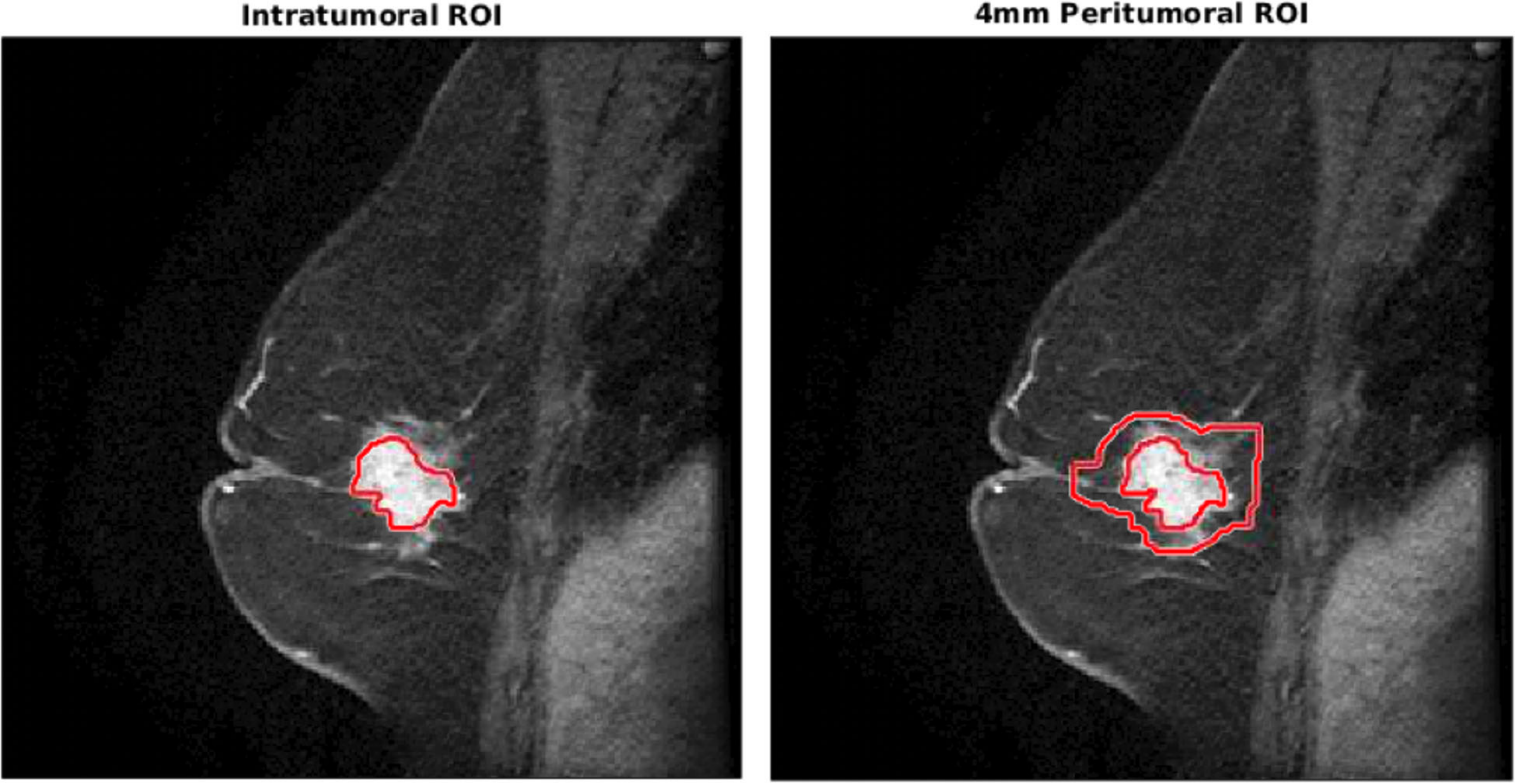
Representative image of intratumoral ROI and peritumoral ROI. Intratumoral ROI was drawn by a radiologist on the first post-contrast image. The peritumoral ROI was generated by dilating the intratumoral ROI by 4 mm

ROIs of the tumor were manually drawn on the first post-contrast image by a radiologist with 11 years of experience. We noted that although manually drawn ROIs can be subjective, an automated convolutional neural network (CNN)-based segmentation was shown to be comparable in radiomics task-based assessment within this cohort [32]. The original ROI was dilated by 4 mm using Matlab v2017b (MathWorks, Natick). This resulted in two regions of interest: one intratumoral ROI and one peritumoral region (0–4 mm). These regions are shown in Fig. 3.

### Feature extraction

#### CR features

Shape features, first order (histogram) features and second order texture features [grey-level co-occurrence matrix (GLCM), neighborhood grey-level different matrix (NGLDM), grey-level run-length matrix (GLRLM), grey-level zone-length matrix (GLZLM)] were extracted following the image biomarker standardization initiative standard [33] using LifeX 3.42 [34]. Laws features [35] were extracted using in-house software written in Matlab v2017b (MathWorks, Natick). Image was quantized to 128 grey levels, and absolute resampling was performed (for intratumoral ROIs, wash-in map: 0 ∼ 640%; wash-out map: − 156 ∼ 100%; SER map: − 1280 ∼ 1280%; for peritumoral ROIs, wash-in map: 0 ∼ 640%, wash-out map: − 540 ∼ 100%, SER map: − 1280 ∼ 1280%). A total of 105 features were extracted. Summary of features is included in Supplemental Table 1. Shape features were only calculated for intratumoral ROI; the remainder of the features were calculated for both intratumoral and peritumoral ROI.

#### DLB features

**Fig. 4:**
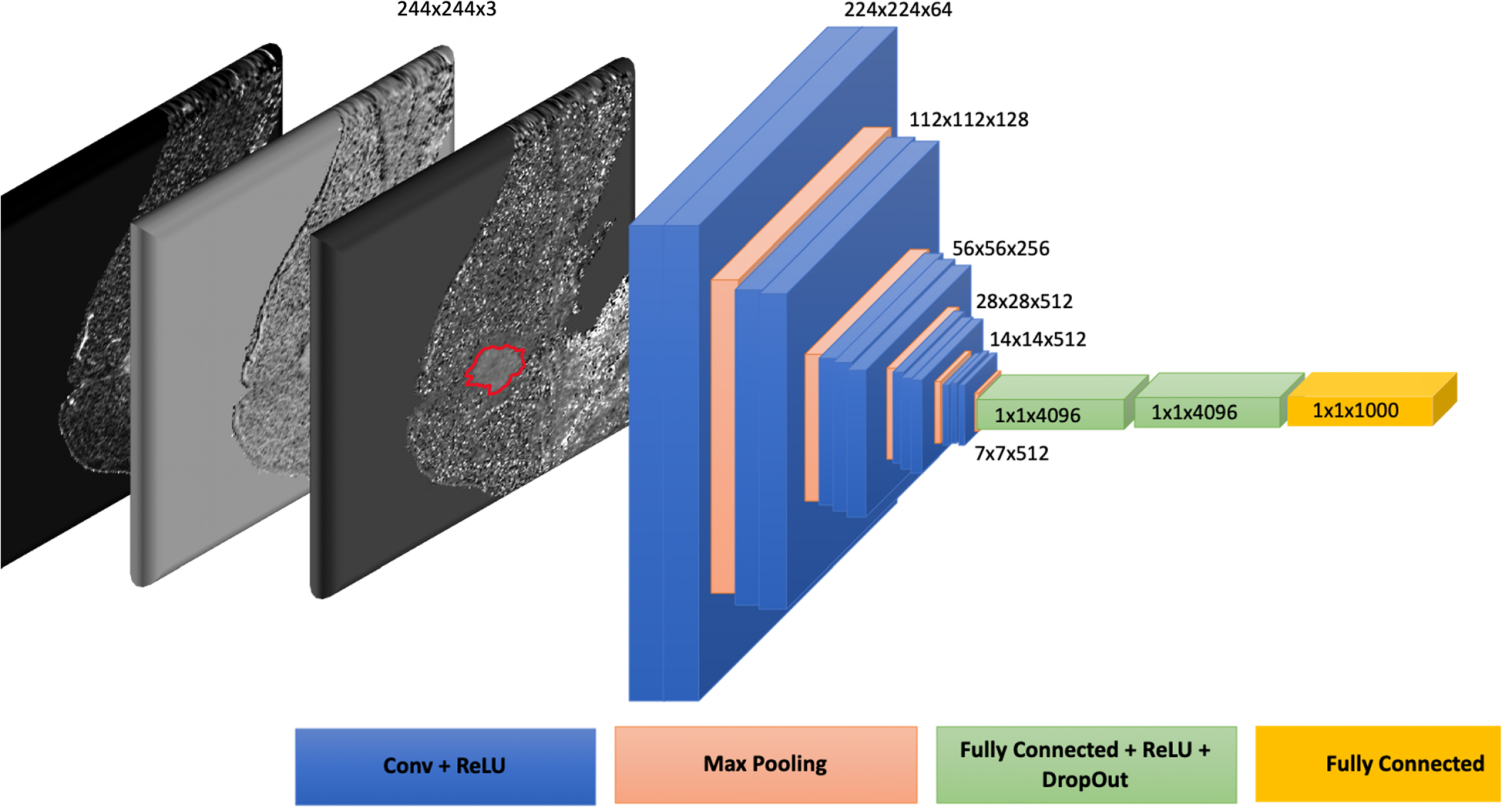
Schematic representation of adapted VGG-16 network for DLB feature extraction. Each layer is defined by corresponding color legend at the bottom of the figure. Input was a 3D volume for a single slice of the three kinetic maps. Features were extracted from the last fully connected layer (yellow), resulting in a feature vector of 1000 features for each 2D slice. Conv: Convolutional layer; ReLu: Rectified linear unit

VGG-16 [36], a pre-trained CNN architecture that is 16 layers deep, was utilized for DLB feature extraction. A schematic representation of the network is shown in Fig. 4.

The image was multiplied by the binary mask of either the intratumoral ROI or peritumoral ROI such that the regions outside of the RIOs were set to zero. Absolute resampling, similar to above, was performed (for intratumoral ROIs, wash-in map: 0 ∼ 640%; wash-out map: − 156 ∼ 100%; SER map: − 1280 ∼ 1280%; for peritumoral ROIs, wash-in map: 0 ∼ 640%, wash-out map: − 540 ∼ 100%, SER map: − 1280 ∼ 1280%). Data were then normalized on a scale from 0 to 1, with 0 being the lowest value and 1 being the highest value referenced above. Then, the resultant image was multiplied by 255 to match the 0–255 range expected by VGG-16.

VGG-16 has a predefined input structure of 224 × 224 × 3. Each 2D slice of our dataset was cropped to 224 × 224. A 3D volume comprised of the Wash-In, Wash-Out, and SER maps for each slice was inputted.

Matlab was used to import VGG-16. The model was not retrained; instead, all layers remained frozen (weights remained the same), and only activations from the last fully connected layer (fc8) were extracted. These were exported as rows, in which each 2D slice had a single row of 1000 features. Given that each ROI has multiple slices, the value in each column was averaged across all slices for that subject.

### Feature reduction

From this point forward, the pipelines for the CR and DLB features are separate and identical.

Standard z-score normalization was used on the training set; z-score is value minus training set mean divided by training set standard deviation. The validation and testing sets were also normalized using the training set mean and standard deviation. Training set was rebalanced using an adaptive synthetic sampling approach; this improves class balance by the creation of new samples from the minority group [37].

Given the high dimensionality of all the extracted features, several steps were performed to remove redundant or non-informative features. Firstly, Mann-Whitney U-test was used to find significantly different features between SLN positive and SLN negative groups; a range of *p*-value thresholds were tested (0.001, 0.005, 0.01, 0.05). Secondly, groups of highly correlated radiomic features were identified (Spearman ρ) and only one representative feature was selected from each correlated group; similarly, several *ρ*-value thresholds were tested (0.75, 0.80, 0.85, 0.90, 0.95). Finally, an optional step of principal component analysis (PCA) was performed to further reduce the feature space; several number of PCA components were tested (20, 40, 60, 80, 100). The optimal thresholds for feature reduction were chosen as those that resulted in the highest validation accuracy of the average of 100 random seeds.

### Feature selection and model creation

The remaining features from the reduction process combined with the clinical features were the input for feature selection process. A logistic regression model was used for the prediction task. The selection of important predictors was performed in the training set using the Least Absolute Shrinkage Selection Operator Regression (LASSO) [38] with 3-fold cross-validation. The selected model was that of minimum cross-validation error plus one standard deviation. To avoid overfitting, the maximum number of the selected features was restricted to 10. These features were then used to establish logistic regression models to predict SLN metastasis. The optimal threshold of the receiver operating characteristic analysis was determined by maximizing the Youden index (YI) in the training set, where the YI is defined as sensitivity + specificity - 1. This threshold was applied to the independent validation and testing datasets. Predictive performance measures tabulated included area under the curve (AUC), sensitivity, specificity, negative predictive value (NPV), positive predictive value (PPV), and accuracy. To avoid the model optimization becoming stuck in a local minimum, the LASSO procedure was repeated 100 times with different seeds. The cross-validation results across all folds were averaged; the model that achieved the highest accuracy in the training set was selected as the prediction model. Additionally, the training set was shuffled each iteration to randomize the cross-validation within the training set, while the independent validation and testing set remained the same.

## Results

### Model incorporating peritumoral region

The primary analysis for this study was the model incorporating intratumoral plus peritumoral (4 mm) features, given that it has been shown to outperform intratumoral features alone in a previous publication [13].

For CR model, a total of 157 features (146 radiomic and 11 clinical) were included in the 3-fold cross-validation LASSO feature selection process. The optimal feature reduction parameters were a *ρ*-value threshold of 0.95, a *p*-value threshold of 0.05, and no PCA. We noted that PCA was also performed for the CR feature reduction pipeline but did not improve the predictive performance of the model. Eight features, including 1 clinical, 2 shape, and 5 texture features, were optimized for this model (Table 1).

**Table 1:** Features chosen by CR pipeline. Features from intratumoral region are denoted by (I) and features from 4 mm peritumoral region are denoted by (P). There was only one clinical feature selected, and the remainder were radiomic features (5 from intratumoral region, 2 from peritumoral region)

Category	Features
**Clinical (*n*** **= 1)**	Lymphovascular invasion
**Shape (*n*** **= 2)**	Compacity (I), Extent (I)
**GLCM (*n*** **= 1)**	Wash Out: Entropy (log2) (I)
**NGLDM (*n*** **= 2)**	Wash Out: Contrast (I) SER: Coarseness (P)
**Laws (*n*** **= 2)**	SER: Energy_8 (I) Skewness_9 (P)

*GLCM* grey-level co-occurrence matrix, *NGLDM* neighborhood grey-level different matrix

For DLB model, the optimal feature reduction parameters were a *ρ*-value threshold of 0.85, a *p*-value threshold of 0.001, and a PCA value of 80. After feature reduction, there were 91 features (80 DLB and 11 clinical) inputted into the feature selection process. For this model, 5 features were finally selected, including 2 clinical (tumor grade and lymphovascular invasion) and 3 DLB features.

**Fig. 5:**
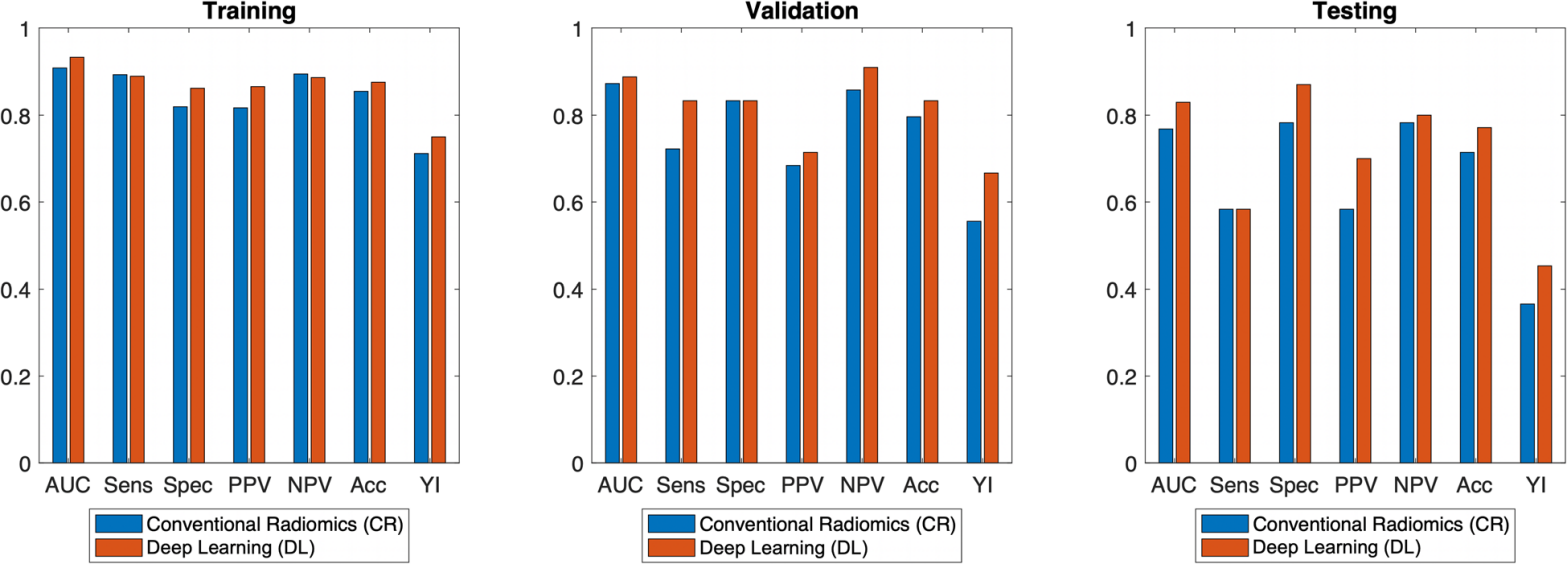
Predictive performance of feature models including intratumoral and 4 mm peritumoral region with CR and DLB pipeline. The DLB pipeline slightly outperformed CR in the validation set. A larger improvement is seen in the testing set of dissimilar resolution. This indicates the DLB pipeline might be more generalizable and insensitive to pixel size differences. Note that the performance of the training set is not used for comparison due to overfitting concerns. *AUC: area under the curve; Sens: sensitivity; Spec: specificity; PPV: positive predictive value; NPV: negative predictive value; Acc: accuracy; YI: Youden index*

Predictive performance metrics are shown in Table 2 and Fig. 5 for the CR and DLB pipelines. In the validation set (i.e., the group with the same resolution as the training set), the DLB model outperformed the CR model [accuracy (CR: 80%, DLB: 83%), YI (CR: 0.56, DLB: 0.67), NPV (CR: 86%, DLB: 91%)]. Furthermore, in the independent testing set of dissimilar resolution meant to evaluate the generalizability of the model to dissimilar condition, the DLB model outperformed the CR model in all metrics [accuracy (CR: 71%, DLB: 77%), YI (CR: 0.37, DLB: 0.45), NPV (CR: 78%, DLB: 80%)]. It is noted that we included the performance of the training set for the completeness of the paper; however, it should not be used for comparison due to overfitting concerns.

**Table 2:** Predictive performance results for intratumoral plus 4 mm peritumoral region for CR and DLB models. Values shown are from the random seed with highest training set accuracy. The DLB pipeline slightly outperformed CR in the validation set of the same resolution as the training set. A larger improvement is seen in the testing set of dissimilar resolution. This indicates the DLB pipeline might be more generalizable and less sensitive to pixel size differences

	**Training (*n* = 109, 37 positive SLNs)**						
	AUC	Sensitivity	Specificity	PPV	NPV	Accuracy	YI
**CR**	0.91	0.89	0.82	0.82	0.89	0.85	0.71
**DLB**	0.93	0.89	0.86	0.86	0.89	0.88	0.75
	**Validation (*n*** **= 54, 18 positive SLNs)**						
	AUC	Sensitivity	Specificity	PPV	NPV	Accuracy	YI
**CR**	0.87	0.72	0.83	0.68	0.86	0.80	0.56
**DLB**	0.89	0.83	0.83	0.71	0.91	0.83	0.67
	**Testing (*n*** **= 35, 12 positive SLNs)**						
	AUC	Sensitivity	Specificity	PPV	NPV	Accuracy	YI
**CR**	0.77	0.58	0.78	0.58	0.78	0.71	0.37
**DLB**	0.83	0.58	0.87	0.70	0.80	0.77	0.45

### Model excluding peritumoral region

As a secondary analysis, models created utilizing clinical features and intratumoral features alone were analyzed.

For CR model, a total of 104 features (93 radiomic and 11 clinical) were fed into the 3-fold cross-validation LASSO feature selection process. The optimal feature reduction parameters were a *ρ*-value threshold of 0.95, a *p*-value threshold of 0.05, and no PCA. In logistic model creation, a maximum of 10 features were included. As discussed in Methods, the random seed with the highest training set accuracy was reported. Finally, there were 10 features chosen, including 2 clinical features, 2 shape features, and 6 texture features (Table 3).

**Table 3:** Features chosen by CR model for intratumoral features only. This model included 2 clinical features and 8 radiomic features

Category	Features
**Clinical (*n*** **= 2)**	Tumor grade Lymphovascular invasion
**Shape (*n*** **= 2)**	Compacity, Extent
**GLCM (*n*** **= 1)**	Wash Out: Entropy (log2)
**GLZLM (*n*** **= 1)**	Wash Out: Long Zone Low Grey Level Emphasis
**Laws (*n*** **= 4)**	WashIn: Skewness_5
	WashOut: Skewness_7
	SER: Energy_4
	Energy_8

For DLB model, the optimal feature reduction parameters were a *ρ*-value threshold of 0.75, a *p*-value threshold of 0.005, and no PCA. After feature reduction, there were 48 features (37 DLB features, 11 clinical) inputted into the 3-fold cross-validation LASSO feature selection process. For this model, 9 features, including 2 clinical features (Tumor grade and Lymphovascular Invasion) and 7 DLB features, were selected.

**Fig. 6:**
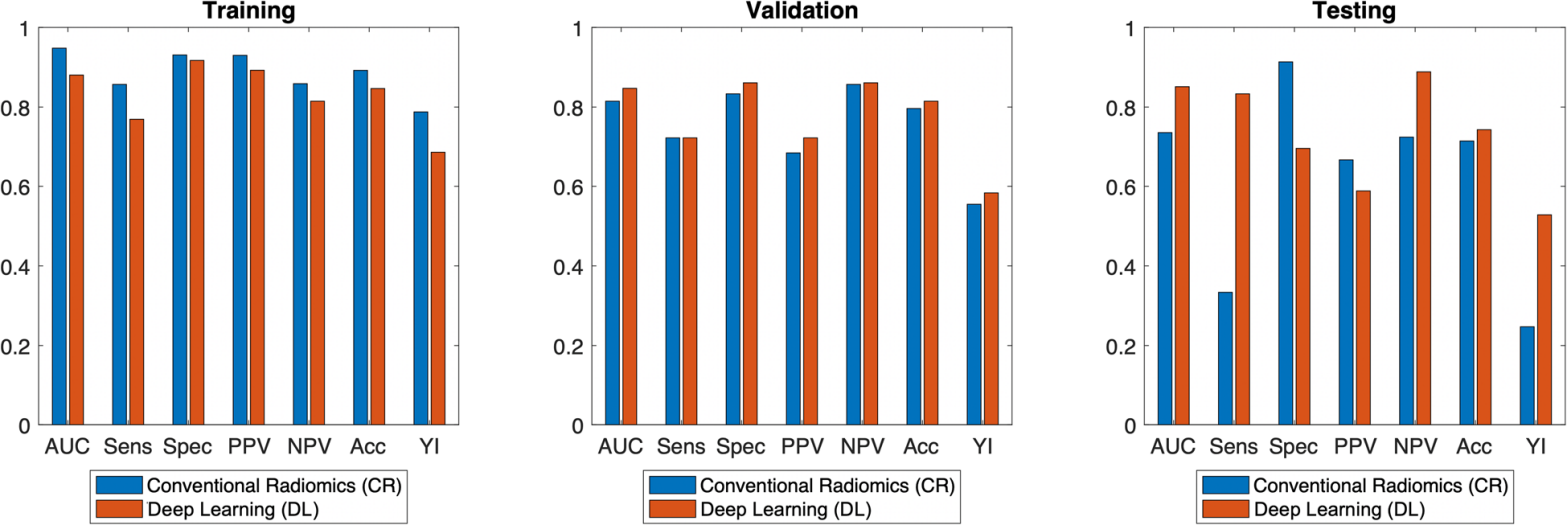
Predictive performance of features including intratumoral region with CR and DLB models. The DLB model performed similarly to CR in the validation set. In the testing set, the DLB model outperformed CR in numerous metrics, including NPV, accuracy and YI. This indicates the DLB model might be more generalizable and less sensitive to pixel size differences. Note that the performance of the training set is not used for comparison due to overfitting concerns. *AUC: area under the curve; Sens: sensitivity; Spec: specificity; PPV: positive predictive value; NPV: negative predictive value; Acc: accuracy; YI: Youden index*

Predictive performance metrics are shown in Table 4 and Fig. 6 for the CR and DLB models. In the validation set, the DLB pipeline performed similarly compared to the CR pipeline [accuracy (CR: 80%, DLB: 81%), YI (CR: 0.56, DLB: 0.58), NPV (CR: 86%, DLB: 86%)]. Furthermore, in the testing set of dissimilar resolution, a similar trend is seen compared, where DLB features outperformed CR features in some metrics [accuracy (CR: 71%, DLB: 74%), YI (CR: 0.25, DLB: 0.53), NPV (CR: 72%, DLB: 89%)]. Similar to above, the performance of the training set should not be used for comparison due to overfitting concerns.

**Table 4:** Predictive performance results for intratumoral region for CR and DLB models. Values shown are from the random seed with highest training set accuracy. The DLB model performed similarly to CR in the validation set. In the testing set, DLB model outperformed CR model in numerous metrics, including NPV, accuracy, and YI. This indicates the DLB model might be more generalizable and less sensitive to pixel size differences

	**Training (*n*** **= 109, 37 positive SLNs)**						
	AUC	Sensitivity	Specificity	PPV	NPV	Accuracy	YI
**CR**	0.95	0.86	0.93	0.93	0.86	0.89	0.79
**DLB**	0.88	0.77	0.92	0.89	0.81	0.85	0.69
	**Validation (*n*** **= 54, 18 positive SLNs)**						
	AUC	Sensitivity	Specificity	PPV	NPV	Accuracy	YI
**CR**	0.81	0.72	0.83	0.68	0.86	0.80	0.56
**DLB**	0.85	0.72	0.86	0.72	0.86	0.81	0.58
	**Testing (*n*** **= 35, 12 positive SLNs)**						
	AUC	Sensitivity	Specificity	PPV	NPV	Accuracy	YI
**CR**	0.74	0.33	0.91	0.67	0.72	0.71	0.25
**DLB**	0.85	0.83	0.70	0.59	0.89	0.74	0.53

## Discussion and conclusions

The results of our study showed that the predictive performance of the DLB model outperformed the CR model in several metrics. More interestingly, these improvements were seen particularly in the independent testing set with dissimilar resolution. This could indicate that DLB features are less sensitive to varying conditions (e.g., pixel size changes) and ultimately result in a more generalizable model.

SLN status prediction has been explored using nomograms; examples include those developed by Memorial Sloan Kettering Cancer Center and MD Anderson, which include age, tumor characteristics (size, grade, type, focality, location), lymphovascular invasion and hormone receptors to predict the likelihood of a diseased, positive SLN. These nomograms have been shown to have a moderate predictive performance [11, 12]. Imaging studies have investigated the use of non-invasive quantitative imaging radiomic biomarkers along with clinical data for prediction of SLN status, with promising results (AUC > 0.8) [9, 13–19]. Specifically, our previously published work has validated a radiomic pipeline of peritumoral region in combination with intratumoral region for the prediction of SLN metastasis [13]. The peritumoral region is of interest because tissue surrounding the tumor may contain valuable information such as angiogenic-lymphangiogenic factors and tumor infiltrating lymphocytes, which have been shown to be related to treatment response [39].

Predictive models based on CR features can be disadvantageous because radiomic features have been shown to be sensitive to changing parameters, such as pixel size alteration [20]. Numerous studies have shown DLB models to outperform CR pipelines. Using MRI, CNN performance has been compared and shown to outperform radiomics for the purposes of breast lesion classification [23] and gene mutations in low grade gliomas [24]. Additionally, the combination of the CR and DLB features was superior in the survival and classification prediction of high-grade gliomas [25, 26]. Our study took one step further, creating an independent testing set of dissimilar resolution, in attempt to identify a particular condition in which DLB features may outperform CR features. Note that the goal of this work is to compare DLB and CR features. Also, given that the radiomic model has shown to outperform the model using clinical characteristics alone in previous study [13], the models presented here are not compared with the clinical only model.

Deep learning can utilize CNNs to extract features by applying convolution layers composed of small-sized fields or kernels, followed by pooling layers to reduce the size of the resultant feature space. Optimizing the kernel to be applied to the image to extract meaningful features is the purpose of training the network. Consequently, the performance of a CNN is dependent on the amount of data it has available to train on. This results in the CNN being more ‘data hungry’ and typically needing a very large sample size, on the order of millions of images, to have optimal performance [40]. Even if collaborating with multiple institutions, acquiring millions of medical images is frequently not feasible, especially in the instances of rare diseases. One way to address this limitation is the utilization of transfer learning. Transfer learning involves the implementation of a pre-trained network that is further fine-tuned with samples specific to a desired task. Transfer learning has been explored for prediction of lymph node metastasis in patients with cervical cancer using MRI (AUC: 0.9) and breast cancer using CT (AUC: 0.8) with promising results [41, 42]. In this work, we used a pretrained VGG model that was originally optimized to classify 1000 types of objects. Thus, the features extracted from the VGG model are expected to be robust as the features were used to differentiate similar shaped objects such as tow trucks and cars, and animals such as ladybugs and crabs. Transfer learning models that further train VGG with new medical imaging data can further manipulate the way features are extracted; whereas our proposed method doesn’t rely on refining the original VGG network and directly utilizes the robust features that have been trained in its original task. Our proposed model uses the last fully connected layer in VGG prior to classification, allowing our DCE images to fully propagate throughout the network for feature extraction. This approach is more readily available compared to transferred learning approach and can be directly incorporated into more traditional radiomics pipeline. Particularly that for studies with relatively small sample size, it is expected to be less prone to overfitting.

Future directions of this study include fine-tuning of the network directly for classification instead of feature extraction [40]. Additionally, incorporation of additional features such as CoLlAGe and wavelet features could be performed for the radiomics pipeline; specifically, it has been shown that these directional-based features have correlated with tumor microenvironment as seen on pathology, such as orientation and densely packed tumor infiltrating lymphocytes [39, 43]. Moreover, other evaluations using different parameters other than in-plane resolution could be performed to further evaluate the generalizability. In general, there also exists the possibility to look specifically at the lymph nodes. This study analyzed the in-breast tumor to predict metastasis to the lymph node. To date, efforts to analyze axillary lymph nodes on MRI has been limited by small axillary lymph node sizes, breast coil sensitivity in regions in the axilla, and exclusion of a majority of the axilla from field of view. Although nodal morphological features on MRI are predictive of malignancy [2, 44], predictive value of contrast enhancement and morphological criteria, such as size and shape of lymph nodes, was found to be controversial [45, 46]. Furthermore, application of this model to more advanced stage breast cancer (e.g., patients undergoing neoadjuvant chemotherapy) or other cancer types (e.g., head and neck cancer) is another potential avenue of study.

## Supplementary Information

**Additional file 1: Supplemental Table 1.** Summary of radiomic features extracted.

## Data Availability

The data that support the findings of this study are available from the corresponding author, CH, upon reasonable request.

## Abbreviations

SLN: Sentinel lymph node
DLB: Deep learning-based
CR: Conventional radiomics
ROI: Region-of-interest
LASSO: Least absolute shrinkage selection operator
ROC: Receiver operating characteristic
DCE: Dynamic contrast enhanced
MRI: Magnetic resonance imaging
TR: Repetition time
TE: Echo time
SER: Signal enhancement ratio
CNN: Convolutional neural network
Conv: Convolutional layer
ReLu: Rectified linear unit
PCA: Principal component analysis
LASSO: Least Absolute Shrinkage Selection Operator Regression
YI: Youden index
AUC: Area under the curve
NPV: Negative predictive value
PPV: Positive predictive value
GLCM: Grey-level co-occurrence matrix
NGLDM: Neighborhood grey-level different matrix
GLRLM: Grey-level run-length matrix
GLZLM: Grey-level zone-length matrix

## References

[1] DeSantis CE, Ma JM, Goding Sauer A, Newman LA, Jemal A (2017). Breast cancer statistics, 2017, racial disparity in mortality by state. CA Cancer J Clin.

[2] Anderson TL, Glazebrook KN, Murphy BL, Viers LD, Hieken TJ (2017). Cross-sectional imaging to evaluate the extent of regional nodal disease in breast cancer patients undergoing neoadjuvant systemic therapy. Eur J Radiol.

[3] Nagar H, Boothe D, Ginter PS, Sison C, Vahdat L, Shin S, Smith M, Chao KSC, Nori D, Hayes MK (2015). Disease-free survival according to the use of postmastectomy radiation therapy after neoadjuvant chemotherapy. Clin Breast Cancer.

[4] Rastogi P, Anderson SJ, Bear HD, Geyer CE, Kahlenberg MS, Robidoux A, Margolese RG, Hoehn JL, Vogel VG, Dakhil SR, Tamkus D, King KM, Pajon ER, Wright MJ, Robert J, Paik S, Mamounas EP, Wolmark N (2008). Preoperative chemotherapy: updates of national surgical adjuvant breast and bowel project protocols B-18 and B-27. J Clin Oncol.

[5] van Nijnatten TJA, Goorts B, Vöö S, De Boer M, Kooreman LFS, Heuts EM (2018). Added value of dedicated axillary hybrid 18F-FDG PET/MRI for improved axillary nodal staging in clinically node-positive breast cancer patients: a feasibility study. Eur J Nucl Med Mol Imaging.

[6] Tonellotto F, Bergmann A, de Souza AK, De Aguiar SS, Bello MA, Thuler LCS (2019). Impact of number of positive lymph nodes and lymph node ratio on survival of women with node-positive breast cancer. Eur J Breast Health.

[7] Ding J, Stopeck AT, Gao Y, Marron MT, Wertheim BC, Altbach MI, Galons JP, Roe DJ, Wang F, Maskarinec G, Thomson CA, Thompson PA, Huang C (2018). Reproducible automated breast density measure with no ionizing radiation using fat-water decomposition MRI. J Magn Reson Imaging.

[8] van Nijnatten TJA, Schipper RJ, Lobbes MBI, Van Roozendaal LM, Vöö S, Moossdorff M (2018). Diagnostic performance of gadofosveset-enhanced axillary MRI for nodal (re) staging in breast cancer patients: results of a validation study. Clin Radiol.

[9] Dong YH, Feng QJ, Yang W, Lu ZX, Deng CY, Zhang L, Lian Z, Liu J, Luo X, Pei S, Mo X, Huang W, Liang C, Zhang B, Zhang S (2018). Preoperative prediction of sentinel lymph node metastasis in breast cancer based on radiomics of T2-weighted fat-suppression and diffusion-weighted MRI. Eur Radiol.

[10] Sollini M, Antunovic L, Chiti A, Kirienko M (2019). Towards clinical application of image mining: a systematic review on artificial intelligence and radiomics. Eur J Nucl Med Mol Imaging.

[11] Bi X, Wang YS, Li MM, Chen P, Zhou ZB, Liu YB, Zhao T, Zhang Z, Wang C, Sun X, Qiu P (2015). Validation of the memorial Sloan Kettering cancer center nomogram for predicting non-sentinel lymph node metastasis in sentinel lymph node-positive breast-cancer patients. Onco Targets Ther.

[12] Nadeem RM, Gudur LD, Saidan ZA (2014). An independent assessment of the 7 nomograms for predicting the probability of additional axillary nodal metastases after positive sentinel lymph node biopsy in a cohort of British patients with breast cancer. Clin Breast Cancer.

[13] Liu CL, Ding J, Spuhler K, Gao Y, Serrano Sosa M, Moriarty M, Hussain S, He X, Liang C, Huang C (2019). Preoperative prediction of sentinel lymph node metastasis in breast cancer by radiomic signatures from dynamic contrast-enhanced MRI. J Magn Reson Imaging.

[14] Choi EJ, Youk JH, Choi H, Song JS (2019). Dynamic contrast-enhanced and diffusion-weighted MRI of invasive breast cancer for the prediction of sentinel lymph node status. J Magn Reson Imaging.

[15] Luo JX, Ning ZY, Zhang SX, Feng QJ, Zhang Y (2018). Bag of deep features for preoperative prediction of sentinel lymph node metastasis in breast cancer. Phys Med Biol.

[16] Ren T, Cattell R, Duanmu HY, Huang P, Li HF, Vanguri R, Liu MZ, Jambawalikar S, Ha R, Wang F, Cohen J, Bernstein C, Bangiyev L, Duong TQ (2019). Convolutional neural network detection of axillary lymph node metastasis using standard clinical breast MRI. Clin Breast Cancer.

[17] Liu J, Sun D, Chen LL, Fang Z, Song WX, Guo DJ, Ni T, Liu C, Feng L, Xia Y, Zhang X, Li C (2019). Radiomics analysis of dynamic contrast-enhanced magnetic resonance imaging for the prediction of sentinel lymph node metastasis in breast cancer. Front Oncol.

[18] Cui XY, Wang N, Zhao Y, Chen S, Li SB, Xu MJ, Chai R (2019). Preoperative prediction of axillary lymph node metastasis in breast cancer using radiomics features of DCE-MRI. Sci Rep.

[19] Han L, Zhu YB, Liu ZY, Yu T, He CJ, Jiang WY, Kan Y, Dong D, Tian J, Luo Y (2019). Radiomic nomogram for prediction of axillary lymph node metastasis in breast cancer. Eur Radiol.

[20] Cattell R, Chen SL, Huang C (2019). Robustness of radiomic features in magnetic resonance imaging: review and a phantom study. Vis Comput Ind Biomed Art.

[21] Afshar P, Mohammadi A, Plataniotis KN, Oikonomou A, Benali H (2019). From handcrafted to deep-learning-based cancer radiomics: challenges and opportunities. IEEE Signal Proc Mag.

[22] Whitney HM, Li H, Ji Y, Liu PF, Giger ML (2020). Comparison of breast MRI tumor classification using human-engineered radiomics, transfer learning from deep convolutional neural networks, and fusion methods. Proc IEEE.

[23] Truhn D, Schrading S, Haarburger C, Schneider H, Merhof D, Kuhl C (2019). Radiomic versus convolutional neural networks analysis for classification of contrast-enhancing lesions at multiparametric breast MRI. Radiology.

[24] Li ZJ, Wang YY, Yu JH, Guo Y, Cao W (2017). Deep learning based Radiomics (DLR) and its usage in noninvasive IDH1 prediction for low grade glioma. Sci Rep.

[25] Han W, Qin L, Bay C, Chen X, Yu KH, Miskin N, Li A, Xu X, Young G (2020). Deep transfer learning and radiomics feature prediction of survival of patients with high-grade gliomas. AJNR Am J Neuroradiol.

[26] Xiao TH, Hua WQ, Li C, Wang SS (2019). Glioma grading prediction by exploring radiomics and deep learning features. Abstracts of the third international symposium on image computing and digital medicine.

[27] Islam M, Ren H (2017) Multi-modal pixelnet for brain tumor segmentation. In: International MICCAI Brainlesion Workshop. Springer, Cham, p 298-308. https://link.springer.com/chapter/10.1007/978-3-319-75238-9_26

[28] Parmar C, Barry JD, Hosny A, Quackenbush J, Aerts HJWL (2018). Data analysis strategies in medical imaging. Clin Cancer Res.

[29] Lawrence S, Giles CL, Tsoi AC, Back AD (1997). Face recognition: a convolutional neural-network approach. IEEE Trans Neural Netw.

[30] LeCun Y, Bengio Y, Arbib MA (1998). Convolutional networks for images, speech, and time series. The handbook of brain theory and neural networks.

[31] Le Cun Y, Boser B, Denker JS, Henderson D, Howard RE, Hubbard W (1989). Handwritten digit recognition with a back-propagation network. Abstracts of the 2nd international conference on neural information processing systems.

[32] Spuhler KD, Ding J, Liu CL, Sun JQ, Serrano-Sosa M, Moriarty M, Huang C (2019). Task-based assessment of a convolutional neural network for segmenting breast lesions for radiomic analysis. Magn Reson Med.

[33] Zwanenburg A, Leger S, Vallières M, Löck S (2016) Image biomarker standardisation initiative. arXiv preprint arXiv:1612.07003

[34] Nioche C, Orlhac F, Boughdad S, Reuzé S, Goya-Outi J, Robert C, Pellot-Barakat C, Soussan M, Frouin F, Buvat I (2018). LIFEx: a freeware for radiomic feature calculation in multimodality imaging to accelerate advances in the characterization of tumor heterogeneity. Cancer Res.

[35] Laws KI (1980). Rapid texture identification. Abstracts of the 0238, image processing for missile guidance.

[36] Simonyan K, Zisserman A (2014) Very deep convolutional networks for large-scale image recognition. arXiv preprint arXiv:1409.1556

[37] He HB, Bai Y, Garcia EA, Li ST (2008). ADASYN: adaptive synthetic sampling approach for imbalanced learning. Abstracts of 2008 IEEE international joint conference on neural networks.

[38] Tibshirani R (1996). Regression shrinkage and selection via the lasso. J R Stat Soc B.

[39] Braman NM, Etesami M, Prasanna P, Dubchuk C, Gilmore H, Tiwari P, Plecha D, Madabhushi A (2017). Intratumoral and peritumoral radiomics for the pretreatment prediction of pathological complete response to neoadjuvant chemotherapy based on breast DCE-MRI. Breast Cancer Res.

[40] Yamashita R, Nishio M, Do RKG, Togashi K (2018). Convolutional neural networks: an overview and application in radiology. Insights Imaging.

[41] Wu QX, Wang S, Zhang SX, Wang MY, Ding YY, Fang J, Wu Q, Qian W, Liu Z, Sun K, Jin Y, Ma H, Tian J (2020). Development of a deep learning model to identify lymph node metastasis on magnetic resonance imaging in patients with cervical cancer. JAMA Netw Open.

[42] Yang XJ, Wu L, Ye WT, Zhao K, Wang YY, Liu WX, Li J, Li H, Liu Z, Liang C (2020). Deep learning signature based on staging CT for preoperative prediction of sentinel lymph node metastasis in breast cancer. Acad Radiol.

[43] Prasanna P, Tiwari P, Madabhushi A (2016). Co-occurrence of local anisotropic gradient orientations (CoLlAGe): a new radiomics descriptor. Sci Rep.

[44] Mattingly AE, Mooney B, Lin HY, Kiluk JV, Khakpour N, Hoover SJ, Laronga C, Lee MC (2017). Magnetic resonance imaging for axillary breast cancer metastasis in the neoadjuvant setting: a prospective study. Clin Breast Cancer.

[45] Kvistad KA, Rydland J, Smethurst HB, Lundgren S, Fjøsne HE, Haraldseth O (2000). Axillary lymph node metastases in breast cancer: preoperative detection with dynamic contrast-enhanced MRI. Eur Radiol.

[46] Mortellaro VE, Marshall J, Singer L, Hochwald SN, Chang M, Copeland EM, Grobmyer SR (2009). Magnetic resonance imaging for axillary staging in patients with breast cancer. J Magn Reson Imaging.

