# Supplementary material for: Preoperative prediction of lymph node metastasis using deep learning-based features
Source: Vis Comput Ind Biomed Art. 2022 Mar 7;5:8. doi: 10.1186/s42492-022-00104-5 (PMC8901808; doi:10.1186/s42492-022-00104-5)
Supplement: Supplementary file 1 — Additional file 1: Supplemental Table 1. Summary of radiomic features extracted. [file 42492_2022_104_MOESM1_ESM.docx]

**Supplemental Table 1** Summary of radiomic features extracted. GLCM: grey-level co-occurrence matrix; NGLDM: neighborhood grey-level different matrix; GLRLM: grey-level run-length matrix; GLZLM: grey-level zone-length matrix.

| Category | Features |
| --- | --- |
| Shape (n = 8) | Volume |
|  | Solidity |
|  | Eccentricity |
|  | Equivalent diameter |
|  | Extent |
|  | Surface area |
|  | Sphericity |
|  | Compacity |
| First order (n = 9) | Minimum |
|  | Maximum |
|  | Mean |
|  | Standard Deviation |
|  | Skewness |
|  | Kurtosis |
|  | Entropy (log10) |
|  | Entropy (log2) |
|  | Energy |
| GLCM (n = 7) | Homogeneity |
|  | Energy |
|  | Contrast |
|  | Correlation |
|  | Entropy (log10) |
|  | Entropy (log2) |
|  | Dissimilarity |
| NGLDM (n = 3) | Coarseness |
|  | Contrast |
|  | Busyness |
| GLRLM (n = 11) | Short Run Emphasis (SRE) |
|  | Long Run Emphasis (LRE) |
|  | Low Grey level Run Emphasis (LGRE) |
|  | High Grey level Run Emphasis (HGRE) |
|  | Short Run Low Grey Level Emphasis (SRLGE) |
|  | Short Run High Grey Level Emphasis (SRHGE) |
|  | Long Run Low Grey Level Emphasis (LRLGE) |
|  | Long Run High Grey Level Emphasis (LRHGE) |
|  | Grey Level Non‐Uniformity (GLN) |
|  | Run Length Nonuniformity (RLN) |
|  | Run Percentage (RP) |
| GLZLM (n = 11) | Short Zone Emphasis (SZE) |
|  | Long Zone Emphasis (LZE) |
|  | Low Grey level Zone Emphasis (LGZE) |
|  | High Grey level Zone Emphasis (HGZE) |
|  | Short Zone Low Grey Level Emphasis (SZLGE) |
|  | Short Zone High Grey Level Emphasis (SZHGE) |
|  | Long Zone Low Grey Level Emphasis (LZLGE) |
|  | Long Zone High Grey Level Emphasis (LZHGE) |
|  | Grey Level Nonuniformity (GLN) |
|  | Zone Length Nonuniformity (ZLN) |
|  | Zone Percentage (ZP) |
| Laws Feature (n = 56)^1^ | Skewness |
|  | Kurtosis |
|  | Entropy (log 10) |
|  | Energy |

^1^Laws features were calculated using 14 filters (L5E5, L5S5, L5R5, L5W5, E5E5, E5S5, E5R5, E5W5, S5S5, S5R5, S5W5, R5R5, R5W5, W5W5) generated from the combinations of 5 kernel vectors: L5 = [1 4 6 4 1], E5 = [–1 –2 0 2 1], S5 = [–1 0 2 0 –1], R5 = [1 –4 6 –4 1], and W5 = [–1 2 0 –2 –1]. L: level; E: edge; S: spot; R: ripple; W: wave.
